# COVID-19 self-protective practices and associated factors among secondary school students in Jimma town, Jimma, Oromia, Southwest Ethiopia

**DOI:** 10.3389/fpubh.2022.1082563

**Published:** 2022-12-23

**Authors:** Zewdie Birhanu, Genzebie Tesfaye, Kasahun Girma Tareke

**Affiliations:** Department of Health, Behavior and Society, Faculty of Public Health, Institute of Health, Jimma University, Jimma, Ethiopia

**Keywords:** COVID-19, Jimma, Ethiopia, self-protection practices, secondary school students

## Abstract

**Background:**

Since there is limited evidence regarding COVID-19 self-protective practices among school students, this study assessed COVID-19 self-protective practices and associated factors among secondary school students.

**Methods:**

A school-based cross-sectional study was conducted in Jimma town, Oromia regional state, Southwest Ethiopia from 25 May 2021 to 10 June 2021. The total sample size was 634, and students were randomly selected from public and private secondary schools. A self-administered questionnaire was used for data collection. The data were entered into Epidata 3.1 and analyzed using SPSS 21.0 software. Descriptive statistics, such as proportion and mean, were computed to describe the findings. The composite index was computed for each dimension. A linear regression analysis was used to identify the predictors of self-protective practice. A local polynomial smoothing graph was done using Stata 12 software to visualize the relationship between a significant variable and an outcome variable.

**Results:**

A total of 576 respondents participated in this study, which made a response rate of 90.85%. The mean score for overall knowledge was 31.40 (SD ±8.65). Knowledge about COVID-19 symptoms and preventive practices had a mean score of 23.93 and 45.96, respectively. The mean scores for perceived vulnerability, severity, benefits, barriers, self-efficacy, and school support were 26.37, 33.21, 43.13, 16.15, 33.38, and 25.45, respectively. The mean score for self-protective practice was 28.38 (SD ±11.04). As perceived benefit (AOR = 0.199, *p* = 0.000, 95% CI: 0.094–0.304), perceived school support (AOR = 0.125, *p* = 0.009, 95% CI: 0.032–0.218), and self-efficacy (AOR = 0.186, *p* = 0.000, 95% CI: 0.102–0.270) increased, COVID-19 self-protective practices also increased and vice versa. However, age (AOR = −0.873, *p* = 0.006, 95% CI = −1.495, −0.251), perceived vulnerability (AOR = −0.107, *p* = 0.021; 95% CI = −0.199, −0.016), and maternal educational status (no formal education) (AOR = −5.395, *p* = 0.000, 95% CI = −7.712 to 3.077) had negatively associated with self-protective practices.

**Conclusion:**

COVID-19 self-protective practice is unsatisfactory. Perceived benefit, perceived school support, and self-efficacy are positively associated with it. However, students' age, perceived vulnerability, and maternal educational status (no formal education) were negatively associated with COVID-19 self-protective measures among secondary school students. The findings underscore that there is a need to conduct risk communications among students. Similarly, awareness creation intervention should target mothers with no formal education.

## Background

The coronavirus disease (COVID-19) is an emerging respiratory infection that causes illnesses ranging from the common cold to severe acute respiratory syndrome. It spreads from human to human through droplets and direct contact (1–5). A person infected with the COVID-19 virus might be asymptomatic (i.e., while spreading the virus) or develop flu-like symptoms, such as fever, runny nose, sneezing, or sore throat, vomiting, diarrhea, nausea, chest tightness, palpitations, dry cough, tiredness, and shortness of breath, immediate medical attention is advised when severe symptoms, such as persistent chest pain or pressure, difficulty breathing, confusion, and bluish face or lips, arise (1–4). For instance, about 80% of reported cases in China had mild-to-moderate diseases, 13.8% had severe diseases, and 6.1% were critical (4–6). Estimates suggest that the median incubation period of the disease ranges from 5 to 6 days, with a range from 1 to 14 days (5, 6).

The World Health Organization (WHO) declared it a global pandemic disease on 11 March 2020. In response, countries around the world have adopted different prevention methods (2, 5, 7), and the public has been instructed to seek information about the disease solely from well-trusted sources and practice protective measures, such as physical distancing, hand hygiene, avoiding touching the eyes, nose, and mouth with unwashed hands (7), and uptake vaccination (8). Similarly, Ethiopia has swiftly implemented several public health measures to stop transmission and prevent the spread of the virus, including partial lockdown, school/university closure, virtual working policy in some sectors, closing borders, mandatory 14 days quarantine for international travelers, declaration of a state of emergency, and others preventive practices (9–11).

The pandemic has become a challenging global burden for all continents affecting humanity's healthcare systems and social, economic, and psychological wellbeing. Low- and middle-income countries are profoundly affected because of deficient medical equipment and fundamental supplies for victims resulting in a disastrous loss of life (12, 13). As of 19 December 2021, COVID-19 has affected over 222 countries and territories, with over 274,583,552 confirmed cases and over 5,368,198 deaths. In Ethiopia, there were 375,810 COVID-19 cases and 6,861 deaths till 19 December 2021 (14).

In addition, it greatly impacts countries' wellbeing and results in increased healthcare costs, unemployment, reduction in remittances, food insecurity, loss of income, school absenteeism, and so on (13). Schools were closed in different countries as safety measures to prevent and stop the spread of the virus (5, 11, 14). As a result, more than 26 million students were affected in Ethiopia, and feeding programs in schools for ~1 million children across multiple regions of the country stopped (15).

However, through gradual assessment, as the impact of COVID-19 is likely to stay over an extended period, the government decided to re-open schools by ensuring safety protocols, such as maintaining physical distance both inside and outside of the classroom; reducing student size per class; consistent use of face masks and frequent hand washing with soap and water; regular screening for symptoms of COVID-19; provision of regular health education, cleaning and disinfection, adequate ventilation, and spacing of desks or grouping of children if required; and availability of necessary resources (5, 11, 14, 15).

The school community, including students, could be a potential amplifier for the transmission of infectious diseases within communities. This is because schools are an important part of the infrastructure where the highest segment of the population spends time. Therefore, maintaining good health in school settings is essential to prevent or reduce the transmission or spread of infectious diseases, such as COVID-19. Existing evidence indicates that young children are often asymptomatic carriers, so they can spread the coronavirus to family and community members without manifesting clinical symptoms. This sustains the invisible transmission of the virus in the community, indicating that COVID-19 transmission in schools is associated with community transmission (5, 6, 15). Moreover, young people and those who spent their time in the school environment are relevant for COVID-19 prevention and control (5, 11, 15). Thus, empowering their knowledge about the disease, access to prevention resources and self-care practices are essential (11, 14, 15).

Although several actions have been implemented to combat the spread of the virus in school settings, little is known about students' practice of COVID-19 prevention measures, especially in the context of Ethiopia. Moreover, evidence was lacking about students' perceptions of COVID-19 and its preventive practices (16). For better implementation of COVID-19 prevention measures in schools, it is critical to assess students' self-protective practices and the determinant factors. Thus, this study assessed COVID-19 self-protective practices and associated factors among secondary school students. Moreover, the findings of the study might be important to review the actions and requirements taken and put in place to prevent the further spread of COVID-19 in schools and in the community and to ensure the safety of children and school staff while at school.

The study was guided by the Health Belief Model (HBM), which is one of the most commonly used behavioral models. It proposes that preventive health behavior is influenced by the following five factors: perceived barriers to making the recommended response, perceived benefits of making the response, perceived susceptibility to the health threat, perceived severity of the threat, and cues to action (17). Accordingly, people take action if they believe that they are susceptible to a disease condition, that it has potentially serious consequences, that the recommended action would reduce the severity of the condition or their susceptibility to it, and that the barriers to taking action are outweighed by benefits. Within the HBM, cues to action can trigger or activate a person's readiness to change health-related behaviors, and self-efficacy is a person's confidence in their ability to change those behaviors (17, 18).

The HBM was selected as the conceptual framework for several reasons. First, the constructs of the model are highly relevant for risk communication and conceptualization of perceptions of severity and vulnerability, which are important motivators for behavioral change or adoption of risk-protective behaviors. Second, given that the coronavirus is an emerging outbreak, public education efforts mostly focus on increasing perceptions of vulnerability to the disease, severity, or consequences due to the disease along with perceptions of self-efficacy and benefits of taking action (17, 18).

In addition to the main constructs of the HBM model, other factors, such as awareness of the knowledge of diseases and preventive measures, exposure to information, and several support factors, can affect people's response to take action (15, 17, 18). Thus, perceived school support was included in the tool to assess the level of school environment support and adherence to preventive practices. This is because schools were reopened considering the school environment needs to be supportive of the school community to prevent COVID-19 preventive practices. Also, schools are infrastructures where large segments of the population, especially the youth and adolescents, spend more of their time and interact with each other. Therefore, there is a need to include this context-based dimension as it contributes to preventing or disseminating COVID-19 infection (10, 12, 13). Similarly, self-efficacy (i.e., an individual's belief in their capacity to execute behaviors necessary to produce specific performance attainments) was added to the additional construct of HBM to assess students' confidence to adhere to COVID-19 preventive practices.

## Methods and materials

### Study design, setting, and period

A school-based cross-sectional study was conducted in Jimma town, Jimma, Oromia regional state, Southwest Ethiopia, from 25 May to 10 June 2021. Jimma town is the largest city in southwestern Oromia and is located 350 km away from Addis Ababa. According to the 2007 population census, the town had an estimated total population of 159,009; 80,897 were boys/men and 78,112 were girls/women (19). The town had 14 secondary schools (eight private and six public). The number of students were 10,720.^1^

#### Study populations

The study population was sampled from secondary school (both public and private) students enrolled in grades 9–12 in the regular program during the 2020/2021 academic year in Jimma town. Students who were absent from school during the study period were excluded.

### Sample size determination

The sample size was determined using a single population proportion formula, *n* = (z_α_/2)^2^p(1 – *p*)/d^2^, where *n* = sample size; *P* = the proportion of school students who exercise appropriate self-care practices toward COVID-19 (i.e., 50%); d: marginal error (5%); and Z α/2: standard normal score at a 95% confidence interval (20, 21). Therefore, n = (1.96)^2*^0.5(1 – 0.5)/ (0.05)^2^ = 384. A design effect of 1.5 was considered to yield a sample size of 576. Considering the 10% non-responsive population, the total sample size was 634.

### Sampling techniques and procedure

Study participants were sampled as follows: first, all secondary schools (grades 9–12) in Jimma town were listed based on information from the Jimma town education office. The list included both public and private schools, which were active during the second semester of the 2021 academic year. These schools were then stratified into two groups: public and private schools. Half of the public schools (three of six) and three of the private schools (three of eight) were randomly selected from each cluster. The number of schools was decided in such a way that it ensured representation. Following this, the total sample size was proportionally allocated to each selected school based on the student size. Within each selected school, further proportional allocation was done by grade level. Finally, an updated list of students was obtained from each grade giving a unique code per each section, and study participants were selected using a simple random sampling technique.

### Variables and measurement

#### Outcome variable

COVID-19 self-care practice (referring to the use of recommended COVID-19 self-protective and safety measures in the school context) (17, 18) was assessed in a comprehensive way using 13 items on a rating scale as always (3), sometimes (2), and never (1) for desirable healthy practices relevant for COVID-19 prevention and safety considering the last 7 days before the survey (14, 15, 22–24). To compute the overall comprehensive practices, all the items were summed up to yield a probable sum score ranging from 0 to 50. Previous studies informed that the items of each construct are summed up and rescaled to (0–100) value for standardization and comparison of the scales using Y = (X – Xmin)/Xrange ^*^ *n*, where Y is the adjusted variable, X is the original variable, Xmin is the minimum observed value on the original variable, Xrange is the difference between the maximum score and the minimum score on the original variable, and n is the upper limit of the rescaled variable. Therefore, first, a separate composite score for each dimension and construct was computed, and the mean value was calculated from the composite score separately after adjusting the score to 50% (24). A higher score indicates higher comprehensive self-care practices and vice versa.

### Explanatory variables

#### Knowledge related to COVID-19

Knowledge-related questions including symptoms and signs (eight items), mode of transmission/risk factors (17 items), and preventions of COVID-19 (seven items), with a response of yes = 1 and no = 0 format (14, 15, 22–24). Then, the overall score number of yes was counted with the higher score indicating higher knowledge (24).

#### Perceived vulnerability and severity

Perceived vulnerability (refers to one's perception of the risk or the chances of contracting COVID-19) (17, 18) was assessed using seven items on a three-point scale (0 = disagree, 1 = not sure, and 2 = agree) and perceived severity (refers to an individual's belief about the seriousness of contracting COVID-19 or the severity of the consequences if one acquires it) was assessed using seven items on a similar rating scale. This three-scale measurement for these constructs was made thinking that it is easier to answer in this way for youth. Then, the score for each construct was computed separately by summing up the item (possible score value, 0–50), with a higher score indicating a higher perceived vulnerability and severity and vice versa (24).

#### Perceived self-efficacy

Perceived self-efficacy (refers to the level of a person's confidence in their ability to successfully perform a behavior) (17, 18) to exercise COVID-19 protective measures was measured using seven items rated as low (1), moderate (2), and high (3). To compute the composite score, the items were summed up with a possible range of 0–50, with a higher score indicating a higher self-efficacy and vice versa (24).

#### Perceived school support/safety

Perceived school safety (the perception that the school environment is safe to protect oneself from COVID-19) (17, 18) measure has assessed the extent to which students feel that necessary support facilities and resources are readily available to them and perceive that the school environment or context is safe to them (15). A total of 20 items were administered to the respondents using a rating scale as always (=3), sometimes (=2), and never (=1). The perceived school support score was computed by summing up the items making a range of possible value scores from 0 to 50. A higher score indicates higher perceived support and vice versa (24).

#### Perceived barriers

Perceived barriers (referring to a person's feelings about the obstacles to performing a recommended health action) (17, 18) were measured by using 13 questions with a three-point scale rated as 0 = disagree, 1 = not sure, and 2 = agree. Accordingly, the probable sum score of the overall perceived barriers to the COVID-19 prevention measure ranged from 0 to 50, with a higher score indicating a higher perceived barrier and vice versa (24).

#### Perceived benefits

Perceived benefits (the desire to avoid illness and the belief that behavior can prevent the illness) (17, 18) were measured by using nine questions with a three-point scale rated as 0 = disagree, 1 = not sure, and 2 = agree. Accordingly, the probable sum score of overall perceived benefits to the COVID-19 prevention measure ranged from 0 to 50, with a higher score indicating a higher perceived barrier and vice versa (24).

#### Cues to actions

Cues to action (stimulus needed to trigger the decision-making process to accept a recommended health action) were measured by using three items with “yes and no” response options. Accordingly, the probable sum score of cues to actions for the COVID-19 prevention measure ranged from 0 to 50. Cues to action items were computed by summing up the item's response value (24).

### Data collection instrument

A structured questionnaire was used to collect the data. The tool was developed by reviewing different literature, including COVID-19 safety and self-protective guidelines and resources (14, 15, 22–24). First, it was prepared in English and translated into Amharic and Afan Oromo languages as respondents are expected to be diverse in language ability. Again, it was translated back to the English version by an independent translator to check for consistency.

The questionnaire consisted of different parts. The first part contained the respondents' (school students) background information. The second part captured knowledge related to COVID-19 (causes, symptoms, transmission, prevention practices, and vulnerable groups). The third part assessed HBM constructs and perceived school support and safety items. The fourth part assessed students' self-protective practices.

### Data collection methods

The data were collected through the self-administrated method. In each school, the principal investigator was closely working with the school director and school teachers, who then facilitated the data collection process, including accessing students' lists, preparing sampling frames, recruiting selected respondents, and administering the questionnaire. Before filling out the questionnaire, adequate orientation, instructions, and guidance were provided to each respondent by data collectors assigned to each school. In the end, the data were checked for completeness, and they took the actions needed (e.g., returning the questionnaire to the respondent for clarifications and corrections).

### Data processing and analysis

After the data collection, data were checked manually for its completeness, and further cleaning work was performed while entering the data into Epidata 3.1 statistical software. Then, they were fed into the Statistical Package for Social Science (SPSS) software version 23 to analyze data. Descriptive statistics were used to describe and summarize the findings, and then the results were expressed as frequency, percentage, mean, and standard deviation as appropriate. The items under each construct were internally reliable with Cronbach's alpha of 0.756 (perceived severity), 0.769 (perceived benefit), 0.703 (perceived barrier), 0.709 (self-efficacy), 0.773 (cues to action), 0.750 (perceived school support), and 0.844 (self-protective practice). All assumptions of linear regression analysis (linearity, normality, multicollinearity, homoscedasticity, etc.) were checked and found to be normal. A simple linear logistic regression model was used to identify candidate variables with a *p* < 0.05 for multiple linear regressions. Then, to identify independent predictors of self-protective behavior, multiple linear regression analysis was performed, and a *p* < 0.05 was used to declare statistical significance. A 95% CI was used to show the degree of association between the independent and response categories of respondents. To visualize the relationship between a significant variable to an outcome variable, a smoothing graph was done using Stata 12 version.

### Data quality assurance and control

The items were checked for face validity by consulting experts in behavioral models. Seven academicians who have research experience in behavioral models were consulted to provide their subjective judgment on the items and operationalization of a construct following indicators, such as representativeness, redundancy of items, clarity, and relevance. Then the tool was pretested on 5% of the sample in secondary school similar contexts, and necessary revisions were made based on the result of the pretest. The item scale was changed from a five-point Likert scale to a three-scale format after the pretest, given that students failed to identify the differences in the five-point Likert scale. Experienced data collectors (bachelor holders) who are fluent in two languages (Amharic and Afan Oromo) were recruited to facilitate the data collection process. Two days of training were given to data collectors on the study purpose, objectives, sampling methods, recruitment of respondents, and questionnaire. The principal investigators were closely supervising the data collection.

### Ethical considerations

The study protocol was approved by an Ethics Review Committee of the Institute of Health, Jimma University, with reference number Postg48-5-2021. Permission was obtained from each school included in the study and the Jimma town education office. Written consent was obtained from each study participant, thoroughly explaining the objectives and purpose of the study. Parents were called to the school and provide written consent for those participants below 18 years. All COVID-19 preventive measures were applied during data collection.

## Results

### Socio-demographic characteristics of participants

A total of 576 respondents participated in this study, which made a response rate of 90.85%. About 296 (51.4%) of the participants were girls/women. The age of the participants ranged from 14 to 22 years, with a mean age of 16.3 (SD ±1.4) years. Regarding religion, the majority (225, 39.1%) of them were orthodox. Almost all (538, 93.4%) participants were single. Regarding educational status, the majority (139, 24.1%) of the students' mothers attended primary school. Similarly, nearly one-fourth of the participants' fathers had attended secondary school (Table 1).

**Table 1:** Sociodemographic characteristics of the study participant, Jimma town, Jimma, Oromia, Southwest Ethiopia, June 2021.

**Variable**	**Categories**	**Frequency**	**Percent**
Sex	Boys/Men	280	48.6
	Girls/Women	296	51.4
Age	14–18 years	460	79.9
	19–22 years	116	20.1
Religion	Orthodox	225	39.1
	Muslims	209	36.3
	Others (Protest, catholic)	142	24.7
Marital status	Single	538	93.4
	Others (Divorce, widowed)	38	6.6
Mothers' occupation	Government employ	123	21.4
	Private employ	138	24.0
	Housewife	246	42.7
	Famers	50	8.7
	Others (not alive)	19	3.3
Fathers' occupation	Government employ	194	33.7
	Private	247	42.9
	daily laborer	22	3.8
	Famers	67	11.6
	Others (alive, )	46	8.0
Mother education	No formal education	113	19.6
	Primary (1–6)	139	24.1
	Junior secondary (7–8)	119	20.7
	Secondary school (9–12)	114	19.8
	University degree and above	91	15.8
Fathers' educations	No formal education	55	9.5
	Primary (1–6)	88	15.3
	Junior secondary (7–8)	89	15.5
	Secondary school (9–12)	139	24.1
	Technical vocation/diploma	113	19.6

### COVID-19 self-protective practices of secondary school student

About 293 (50.9%) always washed their hands frequently with soap and water or alcohol-based hand rub and 307 (53.3%) avoided touching their eyes, nose, and mouth before washing their hands. Approximately 205 (35.6%) and 250 (43.4%) always maintained physical distancing of at least 1 m while in the classroom and outside the classroom, respectively. Moreover, 374 (64.9%) always used face masks in transportation, such as school buses, and 217 (37.7%) of the participant always avoided going to crowded places in schools, such as sports events or student gatherings (Table 2). The cumulative mean score of self-protective practice was 28.38 (median = 28.57; SD ±11.04).

**Table 2:** COVID-19 self-protective practices among secondary school students, Jimma town, Jimma, Oromia, Southwest Ethiopia, June 2021.

**Self-care practice during the seven days before the survey**	**Never**	**Sometimes**	**Always**
	***n*** **(%)**	***n*** **(%)**	***n*** **(%)**
1. Wash hands frequently with soap and water or uses an alcohol-based hand rub	57 (9.9)	226 (39.2)	293 (50.9)
2. Avoided touching eyes, nose, and mouth before washing hands	53 (9.2)	216 (37.5)	307 (53.3)
3. Avoided shaking hands for the greetings	124 (21.5)	164 (28.5)	288 (50.0)
4. Covered cough using the bend of the elbow or a tissue	41 (7.1)	136 (23.6)	399 (69.3)
5. Shared cups, food, or drinks with other students (reversed)	193 (33.5)	203 (35.2)	180 (31.3)
6. Maintained physical distancing of at least 1 meter while in the classroom	154 (26.7)	217 (37.7)	205 (35.6)
7. Maintained physical distancing of at least 2 m while outside the classroom	132 (22.9)	194 (33.7)	250 (43.4)
8. Used facemasks in transportation, such as school buses	49 (8.5)	153 (26.6)	374 (64.9)
9. Avoided going to crowded places in schools, such as sports, student gatherings	177 (30.7)	182 (31.6)	217 (37.7)
10. Used face masks in the classroom	406 (70.5)	130 (22.6)	40 (6.9)
11. Seat alone on one seat in the classroom	168 (29.2)	147 (25.5)	261 (45.3)
12. Staying at home when you were sick or had a common cold or flue	127 (22.0)	153 (26.6)	296 (51.4)
13. Carefully dispose of tissue and disposable items in a closed bin	100 (17.4)	124 (21.5)	352 (61.1)

### Knowledge of participants about COVID-19

The four most mentioned symptoms by respondents were fever (96.7%), dry cough (89.6%), difficulty breathing (86.3%), and sore throat (83.9%). Most of the participants know that COVID-19 spreads through respiratory droplets (95.8%), direct contact with contaminated hands (90.3%), kissing or greetings (87.2%), handshaking (95%), and going to crowded areas (93.2%). Almost all (96%) know that the use of a facemask prevents COVID-19. Similarly, 96.4% of the participants know that avoiding touching their eyes, nose, and mouth before washing their hands is one way of preventing method of COVID-19 and 93.1% of the respondents also know that keeping a physical distance of at least 2 meters is also the other mechanism to prevent the disease (Table 3). The cumulative sum score of items of knowledge about symptoms, transmission, and prevention methods, and overall knowledge was 23.93 (median = 25; SD ±9.61), 25.88 (median = 26.92; SD ±9.53), 45.96 (median = 50; SD ±9.89), and 31.40 (median = 33.33; SD ±8.65), respectively.

**Table 3:** Knowledge of participants about COVID-19 self-protective practice among secondary school students, Jimma town, Jimma, Oromia, Southwest Ethiopia, June 2021.

	**Knowledge items**	**Frequency**	**Percent**
Knowledge of symptoms	Fever	557	96.7
	Dry cough	516	89.6
	Difficult breathing	497	86.3
	Sore throat	483	83.9
	Weakness	297	51.6
	Body ache	196	34.0
	Joint pain	156	27.1
	Diarrhea	58	10.1
Knowledge of transmission of the coronavirus	Through respiratory droplets	552	95.8
	Direct contact with contaminated hands	520	90.3
	Young people like you are not at high risk of getting COVID-19	470	81.6
	Transmit if there is close contact between people	468	81.3
	Transmit by air (airborne)	350	60.8
	I do not know	15	2.6
Knowledge of risk factors for transmissions	Handshaking	547	95.0
	Crowded area	537	93.2
	Persons infected with COVID-19 but have no symptoms cannot transmit the virus to others (reversed)	504	87.5
	Kissing or greetings	502	87.2
	Touching our eyes without having cleaned their hands first	476	82.6
	Inadequately ventilated spaces	415	72.0
	Sharing tables/chairs with other students	234	40.6
	Exchange or share educational materials, such as pens, pencils, books	159	27.6
	Touching our noses without having cleaned their hands first	138	24.0
	Sharing food or drinking	100	17.4
	Touching mouths without having cleaned their hands first	93	16.1
	Sharing toilet	80	13.9
Knowledge of prevention method	Avoiding touching eyes, nose, and mouth before washing hands	555	96.4
	Use facemask	553	96.0
	Not shaking hands	542	94.1
	Washing hands frequently with soap and water	541	93.9
	Keeping a physical distance of at least 2 m	536	93.1
	Cleaning hands using an alcohol-based hand rub	523	90.8
	Avoid going to crowded places, such as bus stations, markets, religious places, sports	503	87.3

### Source of information about COVID-19

**Figure 1:**
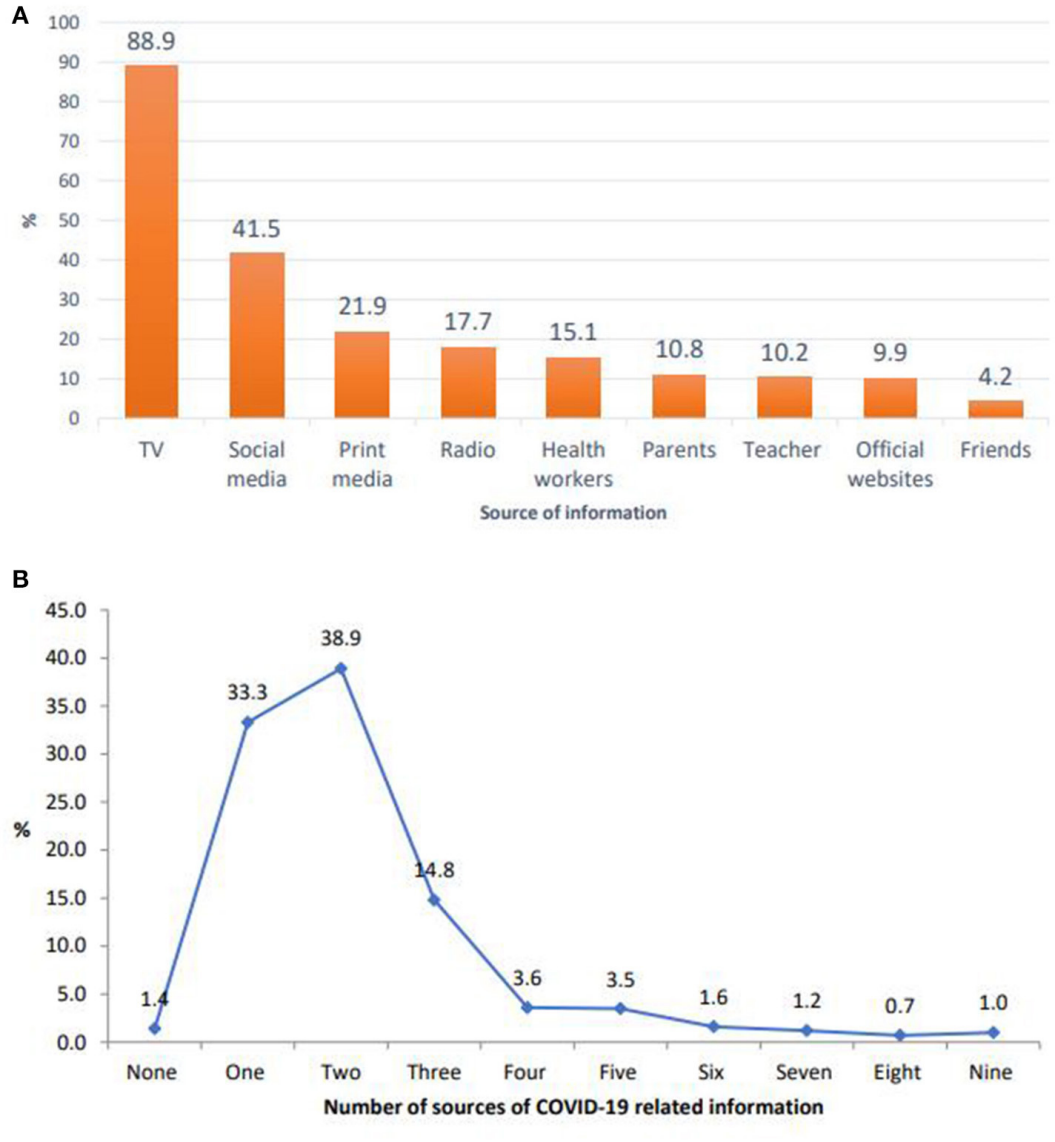
**(A)** Sources of information for COVID-19 among secondary school students in Jimma town, June 2021. **(B)** Number of sources of information for COVID-19 among secondary school students in Jimma town, June 2021.

Electronic media, such as television (88.9%) and social media (41.5%), were the primary source of information, followed by printed materials (21.9%), radio (17.7%), health workers (15.1%), teachers (10.2%), parents (10.8%), and friends/family members (4.2%) (Figure 1A). The average source of information was 2.2 (SD ±1.5), meaning, on average, the respondents received COVID-19-related information from two sources. The majority of the respondents were exposed to one source, two sources, and three sources, which account for 38.9%, 33.3%, and 14.6%, respectively (Figure 1B). The cumulative sum score for the items was 12.23 (median = 11.11; SD ±8.37).

### Perceived vulnerability and severity toward COVID-19

Nearly one-fourth of the participants (31.3%) felt that they were likely to get COVID-19 at school. About 57% of them responded that they would get a COVID-19 infection at school. The majority of the respondents (61.6%) thought that it was less likely to acquire COVID-19 as they are young. The items had a cumulative sum score of 26.37 (median = 26.92; SD ±9.18).

Most (90.6%) of the respondents believed that COVID-19 infection is a severe disease and 93.2% of them believed that COVID-19 is a dangerous disease. More than two-thirds (71.9%) of them believed that COVID-19 is an extremely harmful disease to their family. Only 14.2% of them believed that there is no COVID-19 disease and more than half (55%) of the participants thought that COVID-19 is a severe disease for young people (Table 4). The items had a cumulative sum score of 33.21 (median = 33.33; SD ±8.69).

**Table 4:** Perceived vulnerability and severity toward COVID-19 among secondary school students, Jimma town, Oromia, Southwest Ethiopia, June 2021.

**Items**	**Yes (agree)**		**No (disagree/not sure)**	
**Perceived vulnerability**	***N***	**%**	***N***	**%**
No matter what I do, I am likely to get COVID at school	180	31.3	396	68.8
In many aspects, I am less likely to acquire COVID-19 at school (reversed)	368	63.9	208	36.1
Do you think you will get a COVID-19 infection at school?	330	57.3	246	42.7
Do you think you are at risk of getting COVID-19 because you are a school-going student?	235	40.8	341	59.2
Do you think it is less likely to acquire COVID-19 as you are young?	355	61.6	221	38.4
I think that there is no COVID-19 disease	82	14.2	494	85.8
Only older people are susceptible to COVID-19 (reversed)	463	80.4	113	19.6
**Perceived severity**				
COVID-19 has had a serious impact on my school performance	393	68.2	183	31.8
Do you believe that COVID-19 infection is a severe disease?	522	90.6	54	9.4
Do you think that COVID-19 is a dangerous disease?	537	93.2	39	6.8
Do you believe that COVID-19 is an extremely harmful disease to your family?	414	71.9	162	28.1
Do you think that COVID-19 is a severe disease for young people like you?	317	55.0	259	45.0
I am afraid of COVID-19 because people may discriminate against me if I get it	110	19.1	466	80.9
I do not care about this disease, and I attend my school activities like before	424	73.6	152	26.4

### Perceived barriers and benefits to performing COVID-19 self-protective practice

About 53.1 and 68.8% of respondents had difficulty finding water and soap and getting hand sanitizer in school, respectively. About 465 (80.7%), 479 (83.2%), 470 (81.6%), and 445 (77.3%) of them believed that hand washing, keeping physical distance, wearing a facemask, and avoiding overcrowded place are helpful in preventing COVID-19, respectively. Similarly, about 471 (81.8%) of the respondents believed that washing hands after coughing, sneezing, or doing something is helpful to prevent themselves and their families from COVID-19 (Table 5). Perceived barrier and benefit had cumulative sum scores of 16.15 (median = 13.46; SD ±13.46) and 43.13 (median = 47.06; SD 8.67), respectively.

**Table 5:** Perceived barriers to performing COVID-19 self-protective practice among secondary school students, Jimma town, Oromia, Southwest Ethiopia, June 2021.

**Items**	**Yes (agree)**		**No (disagree/not sure)**	
**Perceived barriers**				
1. It is difficult to find water and soap at school	306	53.1	270	46.9
2. Wearing a facemask is unnecessary	79	13.7	497	86.3
3. It is difficult to get hand sanitizer in school	396	68.8	180	31.3
4. I do not know how to wear a face mask	53	9.2	519	90.1
5. Wearing a face mask makes me look unattractive	97	16.8	479	83.2
6. A face mask is uncomfortable to wear and causes suffocation	229	39.8	347	60.2
7. My family cannot afford to provide me with a face mask regularly	64	11.1	512	88.9
8. I cannot stop shaking hands because my relationships with people become affected	106	18.4	470	81.6
9. I cannot keep physical distancing because my school has crowed	179	31.1	394	68.4
10. I would feel disappointed by my friends for wearing a face mask	240	41.7	336	58.3
11. No one motivates me to wear a face mask	80	13.9	496	86.1
12. No one motivates me to wash my hands regularly	89	15.5	487	84.5
13. No one motivates me to keep a physical distance	105	18.2	471	81.8
**Perceived benefits**				
14. I believe that hand washing is helpful for me to prevent myself from COVID-19	465	80.7	111	19.3
15. I believe that social distancing is helpful for me to prevent myself from COVID-19	479	83.2	97	16.8
16. I believe a face mask prevents me from getting COVID-19 infections	470	81.6	106	18.4
17. When I use a face mask, I feel a sense of responsibility to protect my families and communities	465	80.7	111	19.3
18. Face mask use is helpful to protect others from the virus	503	87.3	73	12.7
19. I believe that avoiding overcrowding places is helpful for me to prevent myself from COVID-19	445	77.3	131	22.7
20. I believe that stopping shaking people's hands is helpful for me to prevent myself from COVID-19	444	77.1	132	22.9
21. I trust the messages my government provides about the pandemic	399	69.3	177	30.7
22. I believe that washing my hands after coughing or sneezing, or doing something is helpful to cure myself and my family	471	81.8	105	18.2

### Self-efficacy about COVID-19 self-protective practices

Nearly, half (51.2%) of the participants had high confidence in washing their hands frequently with soap and water or using an alcohol-based hand rub to kill the COVID-19 virus. Approximately 59.9% of the participants had high confidence in maintaining social distancing to prevent infection with coronavirus. One-third (33.2%) of the participants had high confidence to maintain at least a 1-m distance between themselves and another student to prevent infection with coronavirus. Of note, 54.9% of them had high confidence that they could always use a face mask while going to school (Table 6). The items had a cumulative sum score of 33.38 (median = 33.33; SD ±11.24).

**Table 6:** Self-efficiency of the participants about COVID-19 self-protective practice among secondary school students, Jimma town, Oromia, Southwest Ethiopia, June 2021.

	**Self-efficiency**	**Low**		**Moderate**		**High**	
1	How much are you confident in washing hands frequently with soap and water or using an alcohol-based hand rub kills the virus that causes COVID-19	56	9.7	225	39.1	295	51.2
2	How much are you confident that maintaining social distancing can prevent infection with coronavirus?	34	5.9	197	34.2	345	59.9
3	How much are you confident in avoiding touching eyes, nose, and mouth to prevent infection with coronavirus?	53	9.2	236	41.0	287	49.8
4	How much are you confident in covering your cough/sneezing, using the bend of your elbow or a tissue to prevent the spread of coronavirus?	27	4.7	260	45.1	289	50.2
5	How much are you confident to seek for fever, cough, and difficulty breathing, seeking medical care early help to manage COVID-19?	31	5.4	212	36.8	333	57.8
6	I can maintain at least a 1-m distance between myself and another student to prevent infection with coronavirus in school	63	10.9	322	55.9	191	33.2
7	How much you are confident that you can always use a face mask while going to school?	58	10.1	202	35.1	316	54.9

### Cues to actions

As indicated in Table 7, a minority (35.4%) of the students had seen people get sick from the coronavirus, and 29% of the students' relatives or family members have acquired the coronavirus. Almost all of the students' parents remind them how to protect themselves from the coronavirus while they go to school (Table 7). The items had a cumulative sum score of 30.88 (median = 25.00; SD ±11.60).

**Table 7:** Cues to actions regarding COVID-19 among secondary school students, Jimma town, Oromia, Southwest Ethiopia, June 2021.

**Items**		**Frequency**	**Percentage**
Have you ever seen a person who gets sick from the coronavirus?	No	372	64.6
	Yes	204	35.4
Did any of your relatives or family members acquire the coronavirus?	No	409	71.0
	Yes	167	29.0
Do your parents remind you how to protect yourself from the coronavirus while you go to school?	No	57	9.9
	Yes	519	90.1
Received education at school about the prevention of COVID-19	No	43	7.5
	Yes	533	92.5

### Perceived school support to practice COVID-19 preventive measures among students

Below one-fourth (17.9%) of the students were always crowded when they entered and left the school, and nearly the first quarters (25.7%) of staff and students could move through common spaces without crowding or physical contact. Approximately 45% of the participants always minimized physical contact and close, face-to-face interactions, and 22% of the students have always had a physical distance from other students. More than half (53.6%) of the participants used visual cues (floor markings, posters, etc.) to promote physical distancing (Table 8). The items had a cumulative sum score of 24.45 (median = 25.81; SD ±9.07).

**Table 8:** Perceived school support to self-protect COVID-19 among secondary school students, Jimma town, Oromia, Southwest Ethiopia, June 2021.

	**Items**	**Never**		**Sometimes**		**Always**	
1	Students are crowded when they enter and leave the school (reversed)	151	26.2	327	56.8	98	17.0
2	Staff and students can move through common spaces without crowding or physical contact	103	17.9	325	56.4	148	25.7
3	Physical contact and close, face-to-face interactions are minimized students are spread out as much as possible	51	8.9	266	46.2	259	45.0
4	Physical distancing is practiced by students	145	25.2	304	52.8	127	22.0
5	Visual cues (floor markings, posters, etc.) are in place to promote physical distancing	122	21.2	145	25.2	309	53.6
6	Student gatherings (e.g., events that bring staff and students together outside of regular learning activities) are avoided	140	24.3	168	29.2	268	46.5
7	There is no health school club work on COVID-19 (reversed)	285	49.5	93	16.1	198	34.4
8	Hand cleaning facilities are available and accessible throughout the school and well maintained	122	21.2	245	42.5	209	36.3
9	Signs to remind students to practice regular hand hygiene and good cough etiquette	91	15.8	163	28.3	322	55.9
10	Learning spaces are arranged to maximize the space available and to minimize people directly facing one another	76	13.2	123	21.4	377	65.5
11	My school gives attention to the practice of precautionary measures for the COVID-19 pandemic in the school	85	14.8	164	28.5	327	56.8
12	General cleaning and disinfecting are done every day	406	70.5	96	16.7	74	12.8
13	The school's ventilation system is serviced and operating well.	489	84.9	46	8.0	41	7.1
14	There is an active daily Health Check for students	210	36.5	170	29.5	196	34.0
15	Parents and students are made aware of their responsibilities in COVID prevention	95	16.5	149	25.9	332	57.6
16	Students are reminded to stay home when they are sick	150	26.0	290	50.3	136	23.6
17	Staff wear masks when conducting classroom and outside the classroom	160	27.8	267	46.4	149	25.9
18	Masks are available for those who have forgotten theirs	307	53.3	171	29.7	98	17.0
19	There is educational material at school to guide students practice COVID-19 preventive measures	203	35.2	185	32.1	188	32.6
20	There is health education at school on COVID-19 preventive measures	165	28.6	261	45.3	150	26.0

### Correlation matrix among constricts of HBM and related variables

The correlation among constructs of HBM and other variables is shown in **Table 10**. Accordingly, except for the source of knowledge, knowledge of mode transmissions of COVID-19, perceived severity, and perceived barriers, all the constructs were significantly correlated to self-protective practices (*p* < 0.05). However, perceived vulnerability and cues to action were negatively related to self-reported protective practices (Table 9).

**Table 9:** Correlation matrix among study variables among secondary school students, Jimma town, Oromia, Southwest Ethiopia, June 2021.

	**Self-protective practice**	**P. Vulnerability**	**P. Severity**	**P. Benefit**	**P. Barrier**	**P. S. Support**	**S. Efficacy**	**K. Symptom**	**K. Transmission**	**K. prevention**	**Overall knowledge**	**Cues to action**	**Source of infor- mation**
Self-protective practice	-												
P. Vulnerability	−0.146^**^												
P. severity	−0.081	0.056											
P. benefit	0.256^**^	−0.046	0.203^**^										
P. barrier	−0.092*	0.039	−0.076	−0.231^**^									
P. support	0.164^**^	−0.029	0.017	0.201^**^	−0.043								
S. Efficacy	0.323^**^	−0.142^**^	0.142^**^	0.402^**^	−0.101*	0.185^**^							
K. symptom	0.079	0.025	−0.079	0.080	−0.012	−0.043	0.091*						
K. Transmission	0.041	−0.036	−0.013	0.223^**^	−0.054	0.094*	0.073	0.390^**^					
K. Prevention	0.105*	−0.160^**^	−0.005	0.289^**^	−0.096*	−0.032	0.092*	0.393^**^	0.499^**^				
Overall knowledge	0.084*	−0.058	−0.038	0.245^**^	−0.064	0.031	0.104*	0.715^**^	0.883^**^	0.729^**^			
Cues to action	−0.050	0.042	−0.065	0.077	−0.038	−0.006	−0.042	0.124^**^	0.137^**^	0.184^**^	0.180^**^	–	–
Source of information	0.068	0.023	0.016	0.173^**^	−0.063	−0.099*	0.073	0.339^**^	0.185^**^	0.240^**^	0.304^**^		–

** Correlation is significant at the 0.01 level (two-tailed); ^*^Correlation is significant at the 0.05 level (two-tailed).

### Factors associated with the adherence to COVID-19 self-protective practice among secondary school students

In this study, factors associated with COVID-19 self-protective practices in simple linear regression were age, sex, religion (Orthodox), religion (Muslim), mother occupation (farmer), father occupation (farmer), mother education (no formal), mother education (junior school), mother education (secondary), father education (no formal), perceived vulnerability, perceived benefit, perceived barrier, perceived school support, self-efficacy, knowledge about preventive measures, and overall knowledge with the *p* < 0.05. In the final multiple linear regression analysis, age (in completed years), mother education (no formal education), perceived vulnerability, perceived benefits, perceived school support, and self-efficacy remained significant predictors of secondary school students' practice of COVID-19 self-protective practices at a 95% confidence level and the *p*-value of 0.05.

Accordingly, the age of the respondent, the mother's education (no formal education), and perceived vulnerability were negatively associated with self-protective practices. A unit increase in age would decrease self-protective practice by 0.873 (AOR = −0.873; *p* = 0.006, 95% CI = −1.495, −0.251). Similarly, students whose mothers had not attended would likely decrease self-protective practice by 5.395 (AOR = −5.395; *p* = 0.000, 95% CI = −7.712 to 3.077). A unit increase in perceived vulnerability score would decrease self-protective practices by an average of 0.107 (AOR = −0.107; *p* = 0.021; 95% CI = −0.199, −0.016). However, perceived benefits, perceived school support, and perceived self-efficacy were positively associated was self-care practices; a unit increase perceives benefits, perceived school support, and self-efficacy that would increase self-protective practice by an average of 0.199 (AOR = 0.199; *p* = 0.000, 95% CI: 0.094–0.304), 0.125 (AOR = 0.125; *p* = 0.009, 95% CI: 0.032–0.218), and 0.186 (AOR = 0.186; *p* = 0.000, 95% CI: 0.102–0.270), respectively (Table 10). Moreover, Table 11 indicated the predictive value of significantly associated variables/constructs and model fit information.

**Table 10:** Predictors of COVID-19 self-protective practices among secondary school students, Jimma town, Jimma, Oromia, Ethiopia, June 2021.

**Variables**	**Crude**				**Adjusted**			
	**B**	***P*-value**	**95.0%CI**		**B**	***P*-value**	**95.0%CI**	
			**Lower**	**Upper**			**Lower**	**Upper**
Age in completed years	−1.385	0.000	−2.031	−0.739	−0.873	0.006	−1.495	−0.251^*^
Sex	1.905	0.038	0.102	3.709	0.939	0.277	−0.757	2.635
Religion (Orthodox)	2.837	0.003	0.997	4.676	0.313	0.780	−1.889	2.514
Religion (Muslim)	−2.502	0.009	−4.372	−0.632	−0.791	0.387	−2.587	1.005
Mother occupation (farmer)	−6.462	0.000	−9.631	−3.293	−1.189	0.483	−4.521	2.142
Father occupation (farmer)	−3.239	0.024	−6.048	−0.430	1.318	0.392	−1.706	4.342
Mother education (no formal)	−6.118	0.000	−8.606	−3.630	−5.395	0.000	−7.712	−3.077^*^
Mother education (junior school)	2.363	0.037	0.137	4.589	0.701	0.510	−1.389	2.790
Mother education (secondary)	3.185	0.006	0.929	5.440	0.997	0.391	−1.286	3.279
Father education (no formal)	−4.906	0.002	−7.958	−1.855	−1.089	0.491	−4.190	2.013
Perceived vulnerability	−0.176	0.000	−0.273	−0.078	−0.107	0.021	−0.199	−0.016^*^
Perceived benefit	0.326	0.000	0.225	0.427	0.199	0.000	0.094	0.304^*^
Perceived barrier	−0.102	0.026	−0.193	−0.012	0.027	0.553	−0.062	0.116
Perceived school support	0.199	0.000	0.101	0.298	0.125	0.009	0.032	0.218^*^
Self-efficacy	0.318	0.000	0.241	0.394	0.186	0.000	0.102	0.270^*^
Knowledge about preventive measures	0.117	0.012	0.026	0.208	0.032	0.621	−0.096	0.160
Overall knowledge	0.107	0.045	0.003	0.211	−0.022	0.686	−0.131	0.087

* Significant predictors for self-protective practice.

**Table 11:** Model summary statistics, predictive values constructs, and variables significantly associated with COVID-19 self-protective practices among secondary school students in Jimma town, Jimma, Oromia, Southwest Ethiopia, 2022.

**Model summary**									
**Model**	***R***	*R* ^2^	**Adjusted** *R*^2^	**Std. error of the estimate**	**Change statistics**				
					*R*^2^ **change**	***F*** **change**	**df1**	**df2**	**Sig**. ***F*** **change**
1	0.346^a^	0.120	0.118	10.32010	0.120	78.090	1	574	0.000
2	0.388^b^	0.150	0.147	10.14736	0.031	20.710	1	573	0.000
3	0.416^c^	0.173	0.169	10.01979	0.023	15.683	1	572	0.000
4	0.435^d^	0.189	0.184	9.93045	0.016	11.338	1	571	0.001
5	0.444^e^	0.198	0.190	9.88817	0.008	5.894	1	570	0.016
6	0.454^f^	0.206	0.198	9.84338	0.009	6.199	1	569	0.013

a Predictors: (Constant), self-efficacy.

b Predictors: (Constant), self-efficacy, Mother education-no formal education.

c Predictors: (Constant), self-efficacy, Mother education-no formal education, perceived benefit.

d Predictors: (Constant), self-efficacy, Mother education-no formal education, perceived benefit, perceived vulnerability.

e Predictors: (Constant), self-efficacy, Mother education-no formal education, perceived benefit, perceived vulnerability, perceived school support.

f Predictors: (Constant), self-efficacy, Mother education-no formal education, perceived benefit, perceived vulnerability, perceived school support, age.

**Figure 2:**
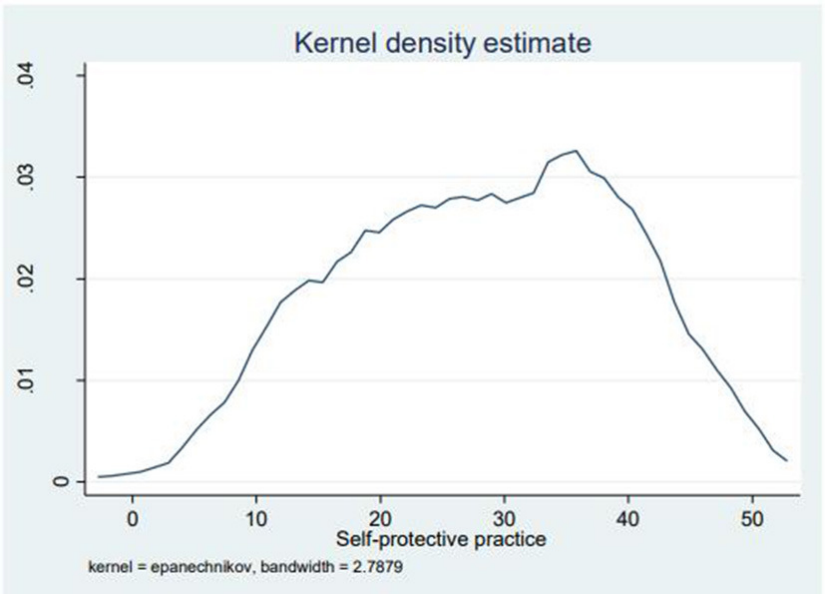
Kernel density estimate of self-protective practices among secondary school students in Jimma town, Jimma, Ethiopia, 2021.

**Figure 3:**
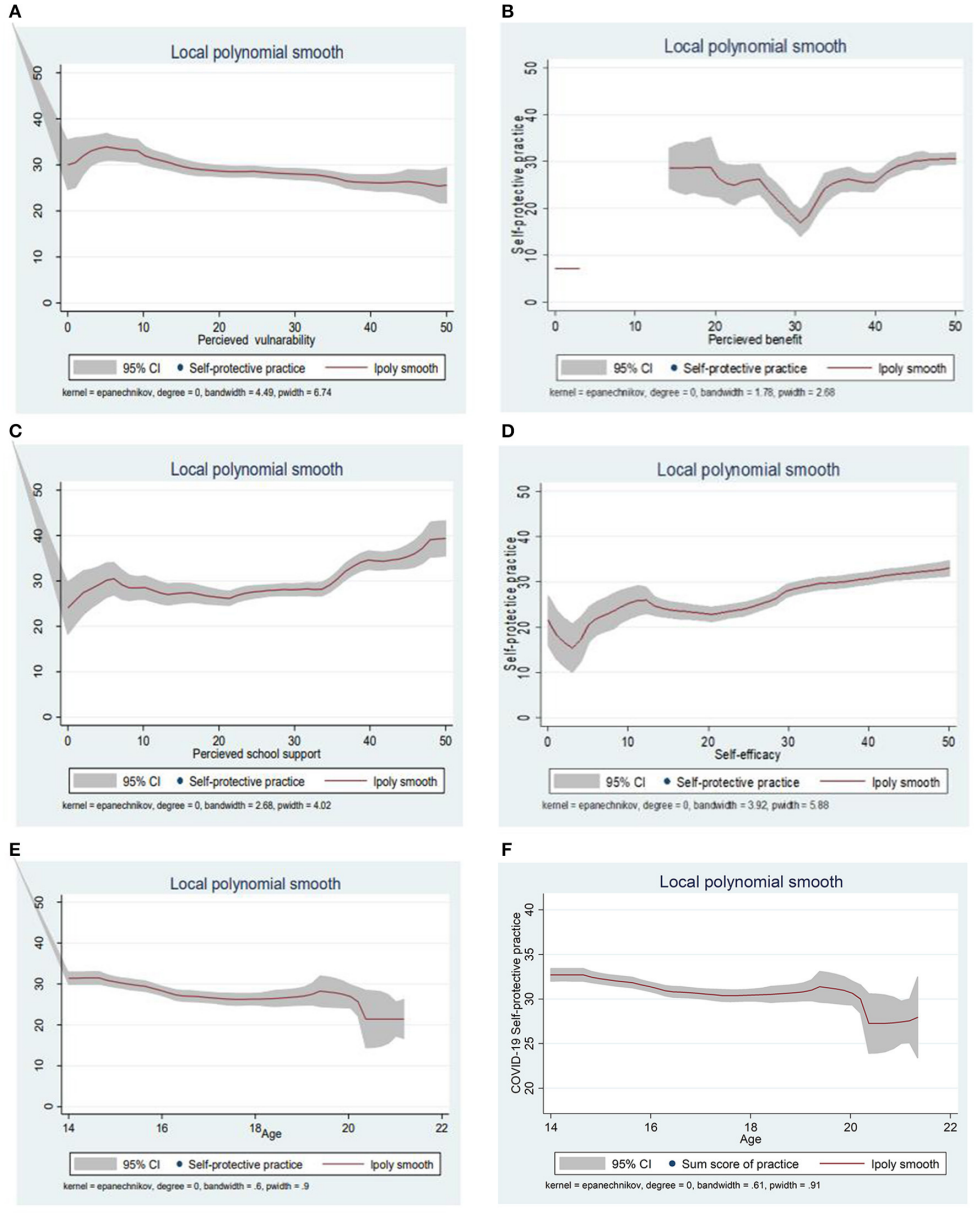
**(A–F)** The local polynomial smoothing to visualize the relationship between dependent and independent variables, which indicates a clear positive and negative (perceived vulnerability) relationship.

In addition, figurative displays were used to show the distributions of self-protective practice scores (possible value: 0–50), indicating that the density of self-protective practice scores was found to be higher around the mid-point, whereas few portions of the respondents scored toward both extremities (Figure 2). A local polynomial smoothing analysis was also done to visualize the relationship between COVID-19 self-protective practices and independent variables. Figure 3A indicates the relationship between COVID-19 self-protective practices and perceived vulnerability (negative relationship) (Figure 3A). However, Figures 3B–F indicate the positive relationship between COVID-19 self-protective practices with perceived benefit, perceived school support, self-efficacy, and age of students, respectively (Figures 3B–F, respectively).

## Discussion

The study found that the cumulative mean score of self-protective practice was 28.38 (SD ±11.04). Accordingly, the age of the respondent, the mother's education (no formal education), and perceived vulnerability were negatively associated with self-protective practices. However, perceived benefits, perceived school support, and perceived self-efficacy were positively associated with it.

The study found that the user adoption of COVID-19 safety and preventive practices among secondary school students was not satisfactory (mean = 28.38). This indicates that strict practice of preventive packages, especially on the use of face masks in the classroom, seating alone (one person per chair), maintaining physical distancing, and hand washing practices were quite low. These might suggest that the school did not pay adequate attention to ensuring COVID-19 school safety protocol, or those young people might be reluctant to use preventive measures. The finding is inconsistent with the expectation that enforcement of school COVID-19 protocol is in place (15). Those young people should practice possible preventive strategies to protect themselves and others from infection (1–7). To combat the pandemic, the school programs and COVID-19 task forces should seriously promote appropriate self-protective behaviors among school students. This finding is slightly higher than the findings of studies conducted elsewhere (15, 16).

The age of the respondent was also negatively associated with COVID-19 self-protective practices. This might imply that, as age increases, they would feel as if they are not at risk of COVID-19 infection or not harmed by it. This underscores that there is a need to conduct risk communications and behavioral change communication interventions about the nature of COVID-19. Similarly, a study conducted in the Egyptian community indicated that people of younger age had good preventive practices (25). In contrast, a study conducted in Iran indicated that age is strongly associated with good preventive practices (26). The difference might happen from geographical or cultural variations.

It was also found that there was a disparity in practicing COVID-19 self-protective measures with respect to maternal education. Students whose mother's education level not attended formal education had decreased self-care practices. This would be because they might not have adequate knowledge about the disease or could not perceive its risk and consequences. Mothers who have higher educational levels have a better understanding of COVID-19 protective methods, and they might be teaching their students about protective measures for COVID-19 and vice versa. This issue also needs greater attention and research on how parents influence and shape COVID-19 behaviors among school students. This is because existing evidence indicates that the more frequent parent–adolescent conversations early in the pandemic predicted increased adherence to HPBs throughout the pandemic (27).

According to the HBM, for an individual to actively perform the behavior, one should hold a belief that they are vulnerable to the disease (17, 18). In the present study, the perceived vulnerability had an adjusted mean score of 26.37 and was negatively associated with self-protective practices. This might imply that those school students did not perceive as they were at risk of COVID-19 infection. Some early studies in Ethiopia also reflected that perceptions of threat (vulnerability and severity) to COVID-19 were low (28, 29). This issue needs further research to investigate why and how the higher perceived vulnerability was associated with decreased self-care practices. Furthermore, there is a need to develop health education programs and conduct risk communication interventions. Similarly, this finding is similar to the findings of studies conducted elsewhere (27).

The study also found that perceived benefit was relatively high (mean = 43.13) and positively associated with self-protective practices. This indicates that those school children correctly appreciated or recognized the effectiveness of COVID-19 preventive measures. Therefore, this underscores the need to strengthen COVID-19 education to enhance perceptions of benefits which is crucial to promoting self-protective behaviors. Similarly, the findings were consistent with other study findings done elsewhere (27, 30).

Similarly, self-efficacy, the personal confidence to take the recommended actions, does not look optimal (mean = 33.38) and is positively associated with self-protective practices. This might imply that self-efficacy is important in practicing preventive measures against COVID-19 disease. Therefore, this underscores the need to strengthen COVID-19 education to enhance perceptions of benefits and build higher self-efficacy crucial to promoting self-protective behaviors. Similarly, the findings were consistent with other study findings done elsewhere (27, 30).

From a practical point of view, school-related public health measures to prevent and minimize calls for addressing the conditions and risk factors that could contribute to the transmission of COVID-19 in school settings and families (15, 31, 32). This requires ensuring access to preventive resources, educational aids, continuous education, and monitoring the adherence to school COVID-19 protocol, and the school should be supportive of students. In this study, we found that the mean score of perceived school support was 25.45 and positively associated with self-protective practices. However, this shows a huge gap in ensuring access to adequate resources needed for COVID-19 prevention, such as water, educational reminder, visual aids, and ensuring classroom facilities that could be COVID-19-sensitive. This is an important finding and the implications for policy and practices, calling for strengthening access to facilities, services, and COVID-19 support infrastructure to establish a safe and supportive environment in the school. Existing evidence also highly recommended it (31, 32).

In principle, knowledge about COVID-19 disease, symptoms of the disease, methods of prevention, and the transmission of COVID-19 infection will increase prevention practices of individuals, and they might be implementing the key messages of the guideline, such as wearing a face mask and proper hand washing practices (5, 7, 12). Inconsistent with this reality, the present study did not find significant associations between knowledge of the diseases and self-protective behaviors. This confirms the notion that knowledge is not a sufficient condition for behavioral change. This calls for the need to go beyond information dissemination and build a knowledge base to influence and address other factors that could affect the practice of self-protective measures.

## Strengths and limitations of the study

This study assessed secondary school students' perceptions and COVID-19 self-protective behaviors based on a theoretical framework (health belief model) and additional constructs, such as school support dimensions, and self-efficacy. The findings might complement other study findings to contribute their own impute for policymakers and for other researchers on controlling the distribution of this pandemic. As a limitation, a three-scale measurement was used, rather than using five-point Likert scale measurements, and respondents specified their level of agreement or disagreement on a symmetric agree–disagree scale for a series of statements, especially for perceived severity and vulnerability items. In addition, there might be limitations, such as social desirability bias (respondents might be made socially acceptable answers, rather than trustful), exaggeration of the answers, or being too embarrassed to reveal private details as the study used a self-administered questionnaire.

## Conclusion

In this study, COVID-19 self-protective practice was unsatisfactory. Perceived benefit, self-efficacy, and perceived school support were positively associated with self-protective measures. However, the age of the respondent, maternal educational status, and perceived vulnerability were negatively associated with it. This underscores the need to strengthen COVID-19 education to enhance perceptions of benefits and build higher self-efficacy along with establishing a safe and supportive school environment to promote self-protective behaviors among school students. Similarly, the findings are for policies and practices, calling for strengthening access to facilities, services, and COVID-19 support infrastructure in the school. Students would benefit from social and behavioral change communication interventions, including risk communication interventions, irrespective of their age, as all people are susceptible to COVID-19 infection. Similarly, awareness creation intervention programs that target maternal educational status should be carried out. Moreover, further research would be important to investigate why and how these variables were associated with decreased self-care practices.

## Data availability statement

The original contributions presented in the study are included in the article/supplementary material, further inquiries can be directed to the corresponding author.

## Ethics statement

The studies involving human participants were reviewed and approved by Jimma University Ethical Review Committee. Written informed consent to participate in this study was provided by the participants' legal guardian/next of kin.

## Author contributions

ZB: conceptualization. ZB, GT, and KGT: data curation, formal analysis, methodology, project administration, validation, visualization, and writing—review and editing. KGT: writing—original draft. All authors have read the manuscript and approved the final version of the manuscript.

## Footnotes

^1^Jimma Town Education Office Report (2021) (unpublished).
